# Adverse and Positive Childhood Experiences and Emotional Regulation Difficulties in a Sample of Incarcerated Men

**DOI:** 10.3390/bs15060828

**Published:** 2025-06-17

**Authors:** Bárbara Maia, Ana Rita Cruz, Olga Cunha

**Affiliations:** 1 HEI-Lab: Digital Human-Environment Interaction Labs, Lusófona University, Campo Grande, 1749-024 Lisboa, Portugal; barbaramaia@outlook.pt (B.M.); p6114@ulusofona.pt (A.R.C.); 2 Psychology Research Center (CIPSI), School of Psychology, University of Minho, Campus de Gualtar, 4710-057 Braga, Portugal

**Keywords:** adverse childhood experiences, positive childhood experiences, emotional regulation difficulties, incarcerated men, prison

## Abstract

Adverse childhood experiences (ACEs) are linked to a higher risk of criminal behaviour, while positive childhood experiences (PCEs) may offer a protective effect by mitigating the negative impact of ACEs. Both ACEs and PCEs play a significant role in shaping emotional regulation. However, research on the influence of PCEs within incarcerated populations remains limited. This study aimed to examine the associations between ACEs, PCEs, and emotional regulation difficulties in a prison sample, and to explore whether PCEs moderate the relationship between ACEs and emotional regulation difficulties in adulthood. The analysis considered both the overall emotional regulation difficulties score and its specific dimensions—strategies, non-acceptance, impulse, goals, awareness, and clarity. The study included 283 men, with a mean age of 40.03 (SD = 11.64), ranging from 19 to 84 years, who were incarcerated in seven prisons in northern Portugal. Data were collected using the Adverse Childhood Experiences Scale, the Benevolent Childhood Experiences Scale, and the Difficulties in Emotional Regulation Scale—Short Form. The results revealed statistically significant positive correlations between ACEs and overall emotional regulation difficulties, as well as with nearly all subscales (strategies, impulse, goals, awareness, and clarity). Conversely, PCEs were significantly negatively correlated with overall emotional regulation difficulties and most subscales (impulse, goals, awareness, and clarity). However, PCEs did not moderate the relationship between ACEs and emotional regulation difficulties. These findings may be influenced by the characteristics of the sample, highlighting the need for further research—preferably longitudinal studies—to better assess the potential moderating role of PCEs. Such research could enhance the effectiveness of prevention and intervention strategies for incarcerated populations.

## 1. Introduction

An individual’s functioning is shaped by life experiences and overall development (Alves et al., 2013; Felitti et al., 1998). Understanding these influences requires examining not only personal characteristics but also the social, cultural, and environmental conditions that shape one’s perception and processing of early experiences (Diener et al., 2018; Pinto et al., 2017). Childhood is a critical period for development, as early experiences can determine adaptive or maladaptive trajectories (Almeida et al., 2021). Due to their vulnerability, children face a heightened risk of adversity, including abuse, neglect, and family dysfunction, which can impair emotional regulation (e.g., Felitti et al., 1998; Marques, 2019).

Conversely, a growing body of research emphasizes that positive childhood experiences (PCEs)—such as consistent affection, safe environments, and meaningful relationships—play a vital role in supporting adaptive functioning and mental health in the face of adversity (Bethell et al., 2019; Gunay-Oge et al., 2020; Narayan et al., 2018, 2020). PCEs can act as protective factors, buffering against the effects of adversity and promoting resilience (Cunha et al., 2024; Gunay-Oge et al., 2020; Sousa et al., 2025). Indeed, several studies have found that PCEs may mitigate the negative impacts of adverse childhood experiences (ACEs) on mental health outcomes (Guo et al., 2022; Sege & Harper Browne, 2017).

Research consistently shows that incarcerated individuals report disproportionately high levels of childhood adversity (Craig et al., 2017), with victimization and neglect often shaping pathways to offending. Moreover, imprisonment itself constitutes a new layer of adversity, often associated with disruptions in biopsychosocial functioning, increased psychopathological symptoms, and emotional regulation difficulties, which can persist post-release (Gonçalves & Gonçalves, 2012; Ranuzi et al., 2020).

Despite growing interest in the protective role of PCEs (e.g., Cunha et al., 2024; Han et al., 2023; Sousa et al., 2025), research examining the coexistence and interplay between ACEs and PCEs—and their combined impact on emotion regulation in incarcerated populations—remains limited. Recent studies have highlighted the importance of understanding how PCEs may buffer the effects of adversity and foster resilience in individuals exposed to cumulative risk (Han et al., 2023), such as those within the prison system. Given this gap, the present study aims to explore how both ACEs and PCEs influence emotional regulation difficulties in adulthood among incarcerated males. Specifically, it investigates the potential moderating role of PCEs in the relationship between ACEs and emotional regulation difficulties. By examining the interaction between risk and protective factors, this study seeks to enhance understanding of developmental trajectories in vulnerable populations and provide empirical evidence to inform therapeutic and correctional interventions.

### 1.1. Adverse and Positive Childhood Experiences

A child gradually develops a sense of self shaped by early attachment relationships and the quality of interactions with caregivers and the surrounding environment (Almeida et al., 2023a). Foundational relational experiences play a critical role in shaping emotional regulation, identity, and adaptation in adulthood (F. M. Silva & Mota, 2018). When these early interactions are marked by consistency, warmth, and security, they foster psychological well-being and long-term adjustment (Almeida et al., 2023a). Conversely, exposure to adversity or disruptions in caregiving relationships has been strongly linked to health problems, emotional deficits, substance use, and behavioural issues (Almeida et al., 2023a; Felitti et al., 1998; Madigan et al., 2023; Senaratne et al., 2024). However, research has predominantly focused on the impact of ACEs, often overlooking the protective role of PCEs. This may stem from a negative bias in human cognition, where attention is drawn more toward adversity, given its significance for survival (Almeida et al., 2021).

The concept of ACEs was introduced by Felitti et al. (1998), encompassing various forms of abuse, neglect, and household dysfunction, including interparental violence, parental substance abuse, mental illness, incarceration, and divorce. Different studies have confirmed the association between ACEs and increased vulnerability to mental health issues, substance abuse, and psychosocial impairments across the lifespan (Anda et al., 2006; Beilharz et al., 2019; Madigan et al., 2023; Malvaso et al., 2018; Senaratne et al., 2024). A meta-analysis by Madigan et al. (2023) found that 22.4% of individuals had experienced at least one ACE, while 16.1% had experienced four or more. The prevalence of four or more ACEs was notably higher among individuals with a history of mental health conditions or substance use disorders. Research consistently demonstrates a high prevalence of ACEs among individuals who have committed crimes (Craig et al., 2017), particularly within incarcerated populations (Alves et al., 2013; Ford et al., 2020; Pessoa & Almeida, 2024). Recent studies indicated that 71% of incarcerated individuals have experienced some form of childhood maltreatment (Almeida et al., 2023b). According to Debowska and Boduszek (2016), emotional abuse (61.7%) and physical abuse (54.3%) are among the most commonly reported adversities within this group. Similarly, Malvaso et al. (2018) found that persistent exposure to physical abuse and neglect was a strong predictor of violent crime. A meta-analysis by Braga et al. (2018) further supports the link between childhood adversity and adult antisocial behaviour. However, despite the strong correlation between ACEs and criminal behaviour, this relationship is not deterministic. Some individuals exposed to ACEs do not develop psychological or behavioural problems later in life (Novak & Fagan, 2022). One potential factor explaining this variability is the presence of PCEs, which may help mitigate the long-term impacts of adversity (Almeida et al., 2023b).

PCEs refer to formative experiences that foster a sense of security, connection, and competence, such as stable relationships with caregivers, safe environments, and opportunities for emotional development (Almeida et al., 2021; Gunay-Oge et al., 2020; Sege & Harper Browne, 2017). The Health Outcomes from Positive Experiences (HOPE) framework (Sege & Harper Browne, 2017) highlights how PCEs support healthy development by interacting with biological, social, and environmental influences (Bethell et al., 2019). It identifies four key types of PCEs: nurturing relationships, safe and stable environments, meaningful social engagement, and development of emotional and social skills (Lewis & McKelvey, 2025). These experiences are often understood as protective factors that, although not universally buffering (e.g., Cunha et al., 2024; Han et al., 2023; Sousa et al., 2025), can mitigate the negative effects of ACEs (Cicchetti, 2013). Importantly, while PCEs do not negate the presence or consequences of ACEs, they can help reduce their impact by equipping individuals with coping strategies to manage adversity more effectively (Duarte et al., 2021; Fabio et al., 2024).

Research highlights the role of PCEs in reducing mental health risks, fostering resilience, and promoting healthy personality development (Bethell et al., 2019; Gunay-Oge et al., 2020). Their sustained presence is associated with improved emotional regulation, lower levels of depression and anxiety, and enhanced self-esteem (Almeida et al., 2023b; Cunha et al., 2024; Kocatürk & Çiçek, 2023). Healthy family ties and adequate parenting practices significantly contribute to adaptive development and the ability to derive meaning from life events (Almeida et al., 2023b; Cunha et al., 2024). Secure early relationships equip children with critical skills to mitigate the effects of adversity (Gunay-Oge et al., 2020), and supportive family dynamics have been shown to protect against the (potential) long-term consequences of ACEs (Narayan et al., 2018; Riedl et al., 2019). For instance, H. Lee and Schafer (2020) found that individuals raised in structured, nurturing environments exhibited greater cognitive functioning and self-control in adulthood.

PCEs also enhance adaptive capacity and resilience (Bethell et al., 2019), with secure attachments and nurturing environments being essential for healthy development (F. M. Silva & Mota, 2018). These experiences are associated with improved interpersonal relationships, job satisfaction, and social engagement later in life (Kocatürk & Çiçek, 2023).

Although research on PCEs is expanding (Cunha et al., 2024; Han et al., 2023; Sousa et al., 2025), there remains a gap in empirical evidence regarding how PCEs and ACEs interact to shape overall functioning in adulthood (Huang et al., 2023). Gaining a deeper understanding of how PCEs contribute to adaptive outcomes in the context of adversity is crucial for informing effective rehabilitation and prevention strategies. This perspective aligns with the HOPE framework (Lewis & McKelvey, 2025; Sege & Harper Browne, 2017) that underscores the importance of not only preventing and mitigating the effects of ACEs but also actively promoting PCEs as essential contributors to lifelong health and well-being (Keane et al., 2025; Lewis & McKelvey, 2025; Sege & Harper Browne, 2017).

### 1.2. Adverse and Positive Childhood Experiences and Emotional Regulation

Emotional regulation is a multifaceted construct that continues to generate scholarly interest and debate, particularly regarding whether it should be viewed as a learned skill, a stable personality trait, or a combination of both (Brackett et al., 2011). Emotional regulation is broadly defined as the ability to adapt to current circumstances by managing emotional responses—enhancing positive emotional states while reducing negative ones (Bridges et al., 2004). According to Gratz and Roemer (2004), emotion regulation encompasses four key dimensions: awareness and understanding of emotions, acceptance of emotional experiences, the ability to control impulses and pursue goal-directed behavior in the context of negative affect, and access to effective emotion regulation strategies. This regulatory process becomes especially relevant when intense emotional reactions interfere with personal goals (Miu et al., 2022). Emotional dysregulation may arise when individuals struggle with limited emotional awareness, difficulty accepting emotional experiences, challenges in maintaining goal-directed behavior, impulsivity, perseveration on maladaptive strategies, and resistance to experiencing discomfort (Cole & Diaz, 2024; Gratz & Tull, 2010).

During childhood, fundamental cognitive processes, such as behavioural response inhibition and emotion regulation, shape more complex psychological functions vital to a better adjustment later in life (V. Lee & Hoaken, 2007). However, experiencing early exposure to adversity can disrupt the development of brain regions critical for emotional processing and regulation (Petchell & Pizzagalli, 2011). Even after the adversity ends, these regions may not fully recover, suggesting long-term disruptions in emotional functioning (Marusak et al., 2015; Petchell & Pizzagalli, 2011). As a result, individuals exposed to ACEs often exhibit difficulties in managing emotional responses, increasing their vulnerability to emotional dysregulation (Almeida et al., 2023a).

Furthermore, ACEs have been associated with specific emotion regulation strategies (Cole & Diaz, 2024; Miu et al., 2022). A meta-analysis by Miu et al. (2022) found that individuals reporting ACEs are more likely to engage in rumination and emotional suppression, and less likely to use adaptive strategies like cognitive reappraisal. Similarly, other studies report associations between ACEs and deficits such as non-acceptance of emotions, poor impulse control, and reduced access to regulation strategies (Cole & Diaz, 2024; Rudenstine et al., 2019). In fact, Cole and Diaz (2024) found that exposure to ACEs was positively associated with all dimensions of emotion regulation difficulties, including emotional awareness, clarity, and goal-directed behavior.

Children who experience abuse may struggle to recognize, express, and understand their emotions, increasing susceptibility to anger, aggression, and other externalizing behaviours (Fox et al., 2015). This is particularly evident in incarcerated populations, where emotional dysregulation is widespread and often linked to difficulties in managing distress, controlling impulses, and relying on maladaptive coping mechanisms (Almeida et al., 2023a; Ford et al., 2020; Neff, 2003).

Moreover, emotional regulation plays a crucial role in the relationship between ACEs and various mental health outcomes (Almeida et al., 2023a). Studies indicate that specific regulation deficits—such as emotional non-acceptance and impulse control difficulties—mediate the impact of ACEs on psychological distress, including depression and anxiety (Cloitre et al., 2019; Rudenstine et al., 2019). In addition, Cole and Diaz (2024) found that, across internalizing disorders, limited access to regulatory strategies emerges as a consistent risk factor, while difficulties with goal-directed behavior and non-acceptance of emotions are especially relevant for depression and emotional distress.

Importantly, the ability to regulate emotions can act as a protective factor, mitigating the negative impact of ACEs and promoting resilience (Morales, 2022). Effective emotional regulation supports adaptive coping, enhances adequate interpersonal functioning, and fosters a greater sense of purpose in life (Almeida et al., 2023a). Emotional dysregulation has been linked to hostility, proactive aggression, and an increased risk of involvement in violent behavior (Almeida et al., 2023a; Ford et al., 2020). ACEs may disrupt emotion processing in ways that impair social cue interpretation (e.g., bias towards negative stimuli), heightening the likelihood of criminal behaviour in adulthood (Marques, 2019). Conversely, PCEs can help children develop emotional regulation skills, reducing the risk of maladjustment or criminal behaviour (Almeida et al., 2023b). According to the HOPE framework (Lewis & McKelvey, 2025; Sege & Harper Browne, 2017), developing social and emotional skills—such as emotional and cognitive self-regulation—is considered a core PCE. While the importance of emotional regulation and the influence of both ACEs and PCEs are well established, further research is needed to fully understand their interactions. Several psychological theories explore how ACEs and PCEs shape individual development.

Developmental Psychopathology Theory (Cicchetti & Toth, 2009) suggests that PCEs are crucial for a child’s adaptive development. Children provided with tools for empathy, resilience, and self-control are better equipped to navigate challenges (Almeida et al., 2021). ACEs, on the other hand, can hinder proper development, increasing vulnerability to behavioural problems (Assche et al., 2020). Initially, this theory linked childhood maltreatment to antisocial behaviour in adulthood (Fox et al., 2015). However, this theory indicated that adversity does not necessarily lead to behavioural problems—developmental trajectories vary based on individual and environmental factors (Toth & Cicchetti, 2013).

The Resilience Theory (Gunay-Oge et al., 2020) defines resilience as the ability to adapt to adversity in a constructive way. It highlights the protective role of positive experiences in mitigating the effects of life’s challenges (Gunay-Oge et al., 2020). Fergus and Zimmerman (2005) categorize protective factors into two groups: skills (inherent to the child) and resources (external support systems such as parental guidance and community support). PCEs foster skills like self-efficacy, self-esteem, and emotional regulation while also reducing the impact of risk factors (Gunay-Oge et al., 2020). A study found that PCEs significantly predict psychological resilience, aligning with this theory (Kocatürk & Çiçek, 2023).

The Transactional Portfolio Model of Resilience (Grych et al., 2015) proposes that the impact of ACEs depends on an individual’s resilience. The more resilient a person is, the better they can adapt to adversity. Their resilience portfolio grows as the individual develops problem-solving and coping strategies (Grych et al., 2015). Since PCEs contribute to psychological resilience (Kocatürk & Çiçek, 2023), they also expand an individual’s resilience portfolio, reducing the negative impact of ACEs (Grych et al., 2015).

When examining the relationship between these constructs, PCEs might buffer the effects of ACEs by fostering self-regulation and emotional regulation, which in turn reduce impulsive and deviant behaviours (Duarte et al., 2021). The absence or scarcity of PCEs increases the likelihood of emotional dysregulation, impulsivity, and eventual involvement in criminal behaviour (Almeida et al., 2023b). However, there is still a lack of empirical research on the relationship between ACEs, PCEs, and emotional regulation difficulties among incarcerated individuals, especially in the Portuguese context.

### 1.3. Current Study

Considering previous literature indicating that both ACEs and PCEs, along with emotional regulation, play a crucial role in shaping an individual’s sense of meaning in life and influencing biopsychosocial outcomes (Almeida et al., 2023b), this study aims to address a gap in the research regarding incarcerated individuals. Given the limited national and international studies examining the relationship between these variables in incarcerated populations, the present study seeks to: (1) investigate relationship between ACEs and emotional regulation difficulties; (2) examine the relationship between PCEs and emotional regulation difficulties; (3) analyze the relationship between ACEs and PCEs; and (4) determine whether PCEs moderate the relationship between ACEs and emotional regulation difficulties in adulthood.

Based on these objectives, and adopting a broad definition of emotional regulation as proposed by Gratz and Roemer (2004), along with previous research findings, the following hypotheses are proposed: (1) ACEs will be positively associated with emotion regulation difficulties, including all dimensions of emotion regulation deficits; (2) PCEs will be negatively associated with emotion regulation difficulties, across all dimensions of emotion regulation deficits; (3) ACEs will be negatively associated with the presence of PCEs; (4) and PCEs will moderate the relationship between ACEs and emotion regulation difficulties, specifically in relation to emotional non-acceptance, impulse control difficulties, and limited access to strategies.

## 2. Method

### 2.1. Sample

This study was conducted using a convenience sample of 283 incarcerated men from seven prisons in Northern Portugal. The inclusion criteria for participation were being male, currently incarcerated, at least 18 years old, and possessing basic reading and writing skills in Portuguese.

As shown in Table 1, participants ranged in age from 19 to 84 years (*M* = 40.03; *SD* = 11.64). The majority had completed either the 6th (*n* = 80; 28.3%) or the 9th grade (*n* = 78; 27.6%), were single (*n* = 147; 51.9%), and belonged to a medium socioeconomic status (*n* = 114; 40.3%). Before their arrest, most participants were employed (*n* = 160; 56.5%).

Additionally, nearly half of the sample had committed non-violent crimes (*n* = 134; 47.3%).

### 2.2. Instruments

The Sociodemographic and Juridical Penal Questionnaire allowed the collection of information regarding age, professional situation prior to imprisonment, socioeconomic status, and marital status, as well as criminal law information such as crime type, or time of imprisonment.

The Adverse Childhood Experiences Scale (ACE; Felitti et al., 1998; Portuguese version S. Silva & Maia, 2008) is a self-report instrument designed to assess the occurrence of ACEs before the age of 18. The original version consists of 77 items, but this study utilized a shortened 17-item version, composed of dichotomous (yes/no) questions. This questionnaire evaluates ten types of childhood adversity: physical abuse, sexual abuse, emotional abuse, emotional neglect, physical neglect, substance abuse in the family environment, divorce or separation of parents or caregivers, imprisonment of a family member, mental illness or suicide of a family member and exposure to domestic violence (DV). The total score is calculated by summing the ten types of abuse, ranging from 0 to 10. Higher scores indicate a greater prevalence of ACEs. Regarding its psychometric properties, the short Portuguese version (S. Silva & Maia, 2008) has demonstrated good reliability and validity. In the present study, the instrument showed a Cronbach’s alpha of 0.91.

The Childhood Benevolent Experiences Scale (BCE; Narayan et al., 2018; Portuguese version Almeida et al., 2021) is a self-report instrument designed to assess the presence of PCEs before the age of 18. It consists of 10 dichotomous (Yes/No) items, categorized into three dimensions: perceived internal and relational security, perceived positive quality of life, and interpersonal support. The total score is calculated by summing the number of affirmative responses, with higher scores indicating a greater prevalence of PCEs (Almeida et al., 2021). The Portuguese version (Almeida et al., 2021) demonstrated good psychometric properties. The present sample revealed a Cronbach’s alpha of 0.70.

The Emotion Regulation Difficulties Scale—Short Form (ERDS-SF; Kaufman et al., 2016; Portuguese version Gouveia et al., 2022) is an 18-item self-report measure assessing difficulties in emotion regulation across different domains. The scale is divided into six subscales, each containing three items: limited access to emotional regulation strategies (strategies), difficulty accepting one’s emotions (non-acceptance), impulse control difficulties (impulse), difficulties engaging in goal-directed behaviours (goals), lack of emotional awareness (awareness), and lack of emotional clarity (clarity). Items are rated on a 5-point Likert scale (1 = almost never; 5 = almost always), with higher scores indicating greater difficulties in emotional regulation. The Portuguese version has demonstrated good psychometric properties (Gouveia et al., 2022). In the present study, the measure showed a Cronbach’s alpha of 0.92.

The Socially Desirable Responses Scale—5 (SDRS-5; Hays et al., 1989; Portuguese version Pechorro et al., 2019) is a five-item self-report scale designed to measure and detect the tendency of participants to provide socially desirable responses, ensuring the validity of the responses. Items are rated on a Likert scale, ranging from “completely true” to “completely false,” with higher scores indicating greater social desirability (Pechorro et al., 2019). In the current study, social desirability was used as a covariate. The Portuguese version of this instrument (Pechorro et al., 2019) demonstrated adequate internal consistency values. In this sample, Cronbach’s alpha was 0.28.

### 2.3. Procedures

The research project was submitted to the Ethics Committee of the [blind for review purposes], where it received approval. Authorization was then requested from the General Directorate of Reintegration and Prison Services, Ministry of Justice, to collect data at various male prisons. After receiving approval, the selected prisons were contacted to schedule data collection. The data was collected from seven prisons in Northern Portugal.

In two prisons, data collection took place in the school setting. Initially, the study’s objectives were explained to the teachers, who were then asked to allocate 30 min of class time for participants to complete the questionnaires. This process was conducted in a group setting, although participants completed the questionnaires individually. In the remaining prisons, data collection was conducted in the library, where prison guards provided a list of individuals who could participate in the study, considering the inclusion and exclusion criteria. The questionnaires were completed individually, though in small groups of 10 to 15 individuals.

During data collection, participants were first asked to read and sign an informed consent form. A brief introduction was given, explaining the study’s objectives, as well as its anonymous, confidential, and voluntary nature. Participants were also informed that they could withdraw at any time without facing any consequences or penalties. No compensation or benefits were provided for participation. The time required to complete the instruments varied from individual to individual, but in most cases, the process took approximately 30 min.

### 2.4. Data Analysis

Data analysis was conducted using IBM SPSS version 29. Descriptive statistics, including mean, standard deviation, minimum and maximum values, frequencies, and percentages, were used to characterize the sample in terms of sociodemographic and juridical-penal variables, as well as total scores and subscales from the instruments.

To test the study hypotheses, Pearson correlation and linear regression analyses, controlled for social desirability, were performed to examine the relationships between ACEs, PCEs, and emotional regulation difficulties (both total score and subscales). Additionally, to assess the potential moderating role of PCEs in the relationship between ACEs and emotional regulation difficulties, six simple moderation analyses (using both the total emotional regulation difficulties score and its subscales) were conducted, controlling for social desirability, using Model 1 of Process Macro 4.2 (Hayes, 2022). The variables were mean-centered prior to conducting the moderation analyses. To reduce the risk of Type I errors resulting from multiple testing (i.e., six moderation analyses), the significance threshold was adjusted to *p* < 0.008.

A statistical power analysis was conducted using G*Power, resulting in a power of 83% (effect size = 0.15, alpha = 0.05), indicating that the sample size was adequate to detect significant effects.

## 3. Results

Table 2 displays the means and standard deviations for the instruments used in this study.

The mean total ACEs score was 2.65 (*SD* = 2.50). Most participants reported experiencing at least one ACE (*n* = 210; 74.2%), while 58.3% (*n* = 165) reported at least two ACEs, 42.8% (*n* = 121) at least three, and 32.9% (*n* = 93) four or more ACEs. The most reported ACEs were exposure to DV (*n* = 204; 72.1%), physical abuse (*n* = 105; 37.1%), exposure to substance abuse (*n* = 99; 35%), emotional abuse (*n* = 89; 31.4%), and divorce or parental separation (*n* = 88; 31.1%).

Regarding PCEs, the maximum score was 10, with a mean of 8.62 (*SD* = 1.74). Nearly all participants reported at least one PCE (*n* = 282; 99.6%), while 98.9% (*n* = 280) reported at least two, 97.8% (*n* = 277), at least three, 96.4% (*n* = 273) at least four, and 80.3% (*n* = 228) eight or more PCEs.

For emotional regulation difficulties (i.e., ERDS-SF), the highest scores were observed on the non-acceptance and strategies scales.

### 3.1. Correlation Analyses

Correlation analyses revealed a significant negative relationship between PCEs and ACEs, indicating that individuals with more ACEs tend to report fewer PCEs (cf. Table 3).

Regarding emotional regulation difficulties and ACEs, statistically significant positive correlations were found between total ACEs score and emotional regulation difficulties total scale, and the strategies, impulse, goals, awareness, and clarity subscales. This suggests that individuals with higher ACEs scores tend to experience greater difficulties in emotional regulation, particularly in employing emotional regulation strategies, controlling impulses, attending to emotional responses, perceiving their emotions accurately, and maintaining goal-directed behaviours (cf. Table 3).

In contrast, PCEs were found to have statistically significant negative correlations with the total score for emotional regulation difficulties and the subscales of impulse, goals, awareness, and clarity. This indicates that individuals with more PCEs tend to exhibit better emotional regulation, and in particular better impulse control, greater attentiveness to emotional responses, improved ability to perceive their emotions, and fewer difficulties in maintaining goal-directed behaviours (cf. Table 3).

Finally, social desirability was positively correlated with ACEs, the overall emotional regulation difficulties scale, and the impulse, goals, awareness, and clarity subscales. This suggests that individuals with greater exposure to ACEs and higher levels of emotional regulation difficulties tend to present higher social desirability. Conversely, social desirability was negatively correlated with PCEs, indicating that those reporting more PCEs tend to exhibit lower levels of social desirability (cf. Table 3).

### 3.2. Regression Analyses

Considering the previous correlational analyses results, six linear regression analyses were performed to determine the relationship between ACEs, PCEs, and emotional regulation difficulties (total scale and strategies, impulse, goals, awareness, and clarity subscales) after controlling for social desirability. Results are displayed in Table 4.

The model predicting overall emotional regulation difficulties was statistically significant, *F*(3, 279) = 11.806, *p* < 0.001. These variables produced an *R*^2^ of 0.113 (*R*^2^ *Adj* = 0.103). Individual analysis revealed that only ACEs (*b* = 1.452, *p* < 0.001, 95% CI [0.757; 2.148]) were positively associated with overall emotional regulation difficulties.

Regarding the prediction of limited access to emotional regulation strategies, the model was statistically significant, *F*(3, 279) = 5.690, *p* < 0.001. The variables produced an *R*^2^ of 0.058 (*R*^2^ *Adj* = 0.048). Individual analysis revealed that only ACEs (*b* = 0.220, *p* < 0.001, 95% CI [0.102; 0.339]) were positively associated with limited access to emotional regulation strategies.

The model predicting impulse control difficulties was also statistically significant, *F*(3, 279) = 9.144, *p* < 0.001; *R*^2^ = 0.090 (*R*^2^ *Adj* = 0.080). ACEs (*b* = 0.221, *p* = 0.006, 95% CI [0.064; 0.377]) and social desirability (*b* = 0.111, *p* = 0.036, 95% CI [0.007; 0.214]) were positively associated with impulse control difficulties.

Regarding the prediction of difficulties engaging in goal-directed behaviours (goals), the model was also statistically significant, *F*(3, 279) = 8.804, *p* < 0.001. These variables produced an *R*^2^ of 0.086 (*R*^2^ *Adj* = 0.077). Only ACEs were positively correlated to difficulties engaging in goal-directed behaviour (*b* = 0.290, *p* < 0.001, 95% CI [0.132; 0.449]).

The model predicting lack of emotional awareness was statistically significant, *F*(3, 279) = 16.035, *p* < 0.001; *R*^2^ = 0.147 (*R*^2^ *Adj* = 0.138). Individual analysis revealed that ACEs (*b* = 0.316, *p* < 0.001, 95% CI [0.170; 0.463]) and social desirability (*b* = 0.136, *p* = 0.006, 95% CI [0.039; 0.233]) were positively associated with lack of emotional awareness.

Regarding the prediction of lack of emotional clarity, the model was also statistically significant, *F*(3, 279) = 15.586, *p* < 0.001. These variables produced an *R*^2^ of 0.144 (*R*^2^ *Adj* = 0.134). Individual analysis revealed that ACEs (*b* = 0.260, *p* < 0.001, 95% CI [0.108; 0.412]) and social desirability (*b* = 0.155, *p* = 0.003, 95% CI [0.054; 0.255]) were positively associated with lack of emotional clarity and PCEs (*b* = −0.233, *p* = 0.037, 95% CI [−0.451; −0.015]) were negatively associated.

### 3.3. Moderation Analyses

Based on the previous analyses, six moderation analyses were conducted using the total score for emotional regulation difficulties and the five subscales (strategies, impulse, goals, awareness, and clarity), while controlling for the effect of social desirability (cf. Table 5).

The analyses conducted revealed no moderating effect of PCEs on the relationship between ACEs and the total scale of emotional regulation difficulties, as well as the subscales of strategies, impulsiveness, sensitivity, clarity, and objectives, while controlling for the effect of social desirability.

## 4. Discussion

The present study aimed to analyze the relationship between ACEs, PCEs, and emotional regulation difficulties (both total scale and subscales), as well as to examine the moderating role of PCEs in the relationship between ACEs and emotional regulation difficulties in adulthood in a sample of incarcerated men. This study contributed to a broader understanding of how PCEs may influence outcomes in the presence of early adversity. Additionally, it seeks to expand the existing body of research on this topic, both nationally and internationally.

The findings revealed that most participants reported experiencing at least one ACE. Although the average ACEs score was relatively low, it is noteworthy that 32.9% of participants reported experiencing four or more ACEs. This aligns with previous research (e.g., Almeida et al., 2023b; Alves et al., 2013; Craig et al., 2017; Ford et al., 2020; Madigan et al., 2023; Pessoa & Almeida, 2024), suggesting that exposure to adversity in childhood increases the likelihood of engaging in delinquent behaviours, ultimately leading to incarceration. This trend can be explained by a child being deprived of a normative and supportive environment crucial for healthy development, which in turn increases their vulnerability to risk factors (Toth & Cicchetti, 2013).

Regarding the most frequently reported ACEs, while physical abuse was common, exposure to domestic violence (DV) was the most frequently reported in this study. This is concerning because DV contradicts the fundamental safety children need from caregivers (Almeida et al., 2021). Such victimization can lead children to normalize violence (Bandura et al., 1961) and has long-term negative effects on emotional processing (Marusak et al., 2015) and increases emotional dysregulation in adulthood (Almeida et al., 2023b).

Despite the high prevalence of ACEs, participants in the present study also reported a high prevalence of PCEs, with most participants (80.3%) reporting eight or more. This finding, consistent with other studies (Almeida et al., 2023a), suggests that incarcerated individuals might report more PCEs than ACEs. Interestingly, 73 participants reported no ACEs, while only one reported no PCEs. This contrasts with most research focusing solely on high ACEs in prison populations (Novak & Fagan, 2022). Several factors might explain these unexpected PCE findings: participants might not recognize some negative experiences as adverse (Bandura et al., 1961); they might underreport ACEs for self-protection (Assche et al., 2020); or the PCE assessment method (simple yes/no) might have influenced responses due to memory issues or lack of careful thought (Narayan et al., 2020).

In examining the relationship between ACEs and emotion regulation difficulties, statistically significant positive correlations were found between ACEs and all emotional regulation subscales, except for non-acceptance of emotional responses. Specifically, higher ACE scores were associated with greater difficulties in engaging in goal-directed behavior, impulse control, emotional awareness, emotional clarity, and accessing effective emotional regulation strategies. These findings suggest that individuals who report more ACEs tend to experience more pronounced emotional regulation difficulties in adulthood. This pattern supports our hypothesis and aligns with previous research demonstrating a link between childhood adversity and impaired emotional regulation (Almeida et al., 2023b). For instance, ACEs have been associated with reduced executive function in early and middle adulthood (Lund et al., 2022) and diminished cognitive flexibility (Kalia & Knauft, 2020), which may hinder the ability to select appropriate emotion regulation strategies in different contexts (Cole & Diaz, 2024). Children who experience maltreatment often struggle to understand and interpret their emotions, making it difficult to identify and respond effectively to intense emotional states. Additionally, ACEs have been shown to disrupt neural reward-processing systems, potentially contributing to challenges in maintaining goal-directed behavior (Novick et al., 2023). The emotional dysregulation caused by ACEs may increase vulnerability to maladaptive behaviours, including criminal activity (Marques, 2019). However, no significant association was found between ACEs and non-acceptance of emotional responses, which contrasts with previous studies suggesting that childhood adversity may lead to negative beliefs about the acceptability of one’s emotion (Diaz & Eisenberg, 2015; Cole & Diaz, 2024). One possible explanation is that the non-acceptance of emotions may be influenced more heavily by individual differences in temperament, cultural or familial emotional norms, or later-life experiences, rather than ACEs alone. It is also possible that some individuals develop compensatory emotional coping mechanisms or resilience strategies that mitigate non-acceptance despite a history of adversity. Additionally, non-acceptance of emotional responses appears to be mostly associated with internalizing disorders, relevant for depression and emotional distress (Cole & Diaz, 2024), and individuals in our sample, although not assessed for internalizing problems, appear to demonstrate rather externalizing behavioral problems.

Similarly, when examining the relationship between PCEs and emotional regulation difficulties, statistically significant negative correlations were observed with overall emotion regulation difficulties, as well as with specific domains, such as difficulties engaging in goal-directed behavior, impulse control, lack of emotional awareness, and lack of emotional clarity. These results indicate that individuals who reported more PCEs tended to experience fewer difficulties in emotional regulation. Although PCEs were not significantly correlated with non-acceptance of emotional responses or limited access to emotional regulation strategies, our findings support the initial hypothesis. PCEs may help create environments that promote the development of self-regulation, self-control, and self-efficacy (Almeida et al., 2023b; Gunay-Oge et al., 2020), equipping individuals with better tools to manage emotional challenges (Almeida et al., 2021). However, when both PCEs and ACEs were included in the same model, PCEs were negatively associated only with lack of emotional clarity and did not significantly influence the other dimensions of emotion regulation or overall emotion regulation difficulties in the presence of ACEs. These findings highlight the potent and potentially overriding impact of ACEs on emotional regulation, suggesting that the protective effects of PCEs may be limited when ACEs are also present (e.g., Cunha et al., 2024; Han et al., 2023).

Regarding the correlations between ACEs and PCEs, previous studies have found statistically significant negative correlations between the two variables (Almeida & Costa, 2023; Almeida et al., 2021; 2023a; Bethell et al., 2019; Narayan et al., 2018), suggesting that individuals who experience a greater number of PCEs tend to report fewer ACEs, and vice versa. The findings of the present study confirm our hypothesis and align with previous literature, as ACEs were once again found to be negatively associated with PCEs. Indeed, research on PCEs suggests that adults who report a higher number of PCEs tend to report fewer ACEs. This finding indicates that children facing adversity may have limited access to supportive relationships and protective resources (Morris & Hays-Grudo, 2023).

In the present study, and contrary to our hypothesis, no moderating effect of PCEs was found in the relationship between ACEs and emotional regulation, even when controlling for social desirability. Existing research on the interplay between ACEs and PCEs and their combined impact on emotional regulation remains limited (Huang et al., 2023). According to Duarte et al. (2021), while PCEs do not negate the presence or consequences of ACEs, they are believed to mitigate their impact, potentially serving a “buffering” role. However, the lack of moderation effect observed in this study may be attributed to specific characteristics of the participant group (i.e., individuals in prison). Alternatively, given that this relationship is not yet well-established in the literature, it is possible that such a moderating effect does not exist. Indeed, recent systematic reviews on PCEs found that the presence of positive experiences often does not moderate the effects of adversity (Cunha et al., 2024; Han et al., 2023), highlighting the need for further research on this topic across diverse populations.

## 5. Practical Implications and Limitations

The literature consistently highlights the profound impact of ACEs on an individual’s development. Therefore, studying ACEs, PCEs, and emotional regulation is essential for advancing knowledge in developmental, forensic, and clinical psychology (Almeida et al., 2023a). Given that this study focuses on the prison population, its practical implications emphasize the need for offender rehabilitation strategies that incorporate different levels of prevention. The findings of this study underscore the urgency of implementing prevention programs for children and adolescents. These programs should aim to reduce the adversity, foster positive experiences, increase emotion regulation, and ultimately lower the risk of engagement in criminal behaviour (Almeida et al., 2023a). Additionally, involving parents in positive parenting programs is crucial. Equipping them with effective strategies and skills can provide the necessary support for their children’s healthy development (Gunay-Oge et al., 2020). Supporting parents, caregivers, and professionals through ACEs prevention initiatives while promoting PCEs can significantly mitigate emotional dysregulation, behaviour issues, and criminal involvement (Grady et al., 2017; Sahle et al., 2020). The training of health and justice professionals is also a key priority. These professionals should receive specialized training in trauma-informed care, emotional regulation, and empathy to better understand the complex life trajectories of incarcerated individuals.

For the prison population specifically, psychological interventions such as trauma-focused therapy can play a vital role in helping individuals process and heal from ACEs (Fox et al., 2015). Additionally, integrating evidence-based programs to enhance emotional regulation skills, such as Cognitive-Behavioural Therapy, Dialectic Behavioural Therapy, or Mindfulness-based approaches, could significantly benefit incarcerated individuals (Ford et al., 2020). Beyond psychological interventions, fostering a positive prison environment is also critical. Expanding educational and vocational training opportunities can enhance individuals’ sense of purpose and personal growth. Moreover, promoting recreational, sports, and cultural activities within prison settings could contribute to the development of positive experiences and reinforce emotional regulation.

Although the contributions of this study are significant, some limitations should be mentioned. A primary limitation relates to the sample: as a convenience sample consisting exclusively of incarcerated males from various prisons in the northern region of Portugal, the findings cannot be generalized to the broader prison population. Another limitation concerns the data collection method, which relied on self-report instruments. This approach may have influenced the results due to the potential for social desirability (Almeida et al., 2021). Despite the inclusion of a social desirability measure in this study, the low Cronbach’s alpha coefficients prevent us from drawing reliable conclusions. We hypothesized possible explanations for the low reliability coefficient, namely, it may be due to the low number of items of the scale or the fact that the scale was not originally developed for offenders’ samples. Moreover, the word formulation of the items might not be adequate for the target population, and, although less probable, due to the limited number of items, the scale can be measuring more than one facet of social desirability.

Additionally, the length of the questionnaires may have contributed to participant fatigue and disengagement, potentially affecting response accuracy. Furthermore, the instruments used to assess ACEs and PCEs measure only their occurrence, without accounting for severity, intensity, or frequency (Anda et al., 2020). As a result, important indicators that could provide deeper insights into the impact of these experiences remain unmeasured, representing another significant limitation. In addition, the cross-sectional design of this study poses constraints, as longitudinal research would be more effective in capturing the evolving relationship between ACEs, PCEs, and emotional regulation over time. The lack of a longitudinal approach may limit the depth of the findings and the robustness of the conclusions drawn. For future research, it is crucial to continue exploring ACEs, PCEs, and emotional regulation with a more representative sample, ideally encompassing incarcerated individuals from all regions of the country and addressing samples of youth involved in the justice system. Although challenging, future studies could move beyond self-report measures and incorporate in-depth individual interviews with prisoners to gain a more nuanced understanding of the severity of their experiences and capture aspects that standardized instruments may overlook. Another valuable direction for future research would be to examine these constructs within specific categories of crime or patterns of aggressiveness among offenders with community measures to determine whether significant differences exist. Additionally, further investigation into the role of PCEs in the relationship between ACEs and emotional regulation is needed to either confirm or refute their proposed moderating effect.

## 6. Conclusions

In conclusion, the first three hypotheses were confirmed, demonstrating that ACEs are associated with emotional regulation difficulties, while PCEs contribute to greater emotional regulation in adulthood, and there was a negative association between PCEs and ACEs. However, the fourth hypothesis was not supported, as no moderating role of PCEs in the relationship between ACEs and emotional regulation difficulties was found. Despite this, the study highlights important practical implications for prevention, intervention, and offender rehabilitation, given the high prevalence of adversity in this population. Once again, it reinforces the significant impact of childhood experiences—both positive and adverse—on individual development. Finally, it underscores the need for continued research exploring these variables through different methodologies and in diverse populations, including young offenders.

## Figures and Tables

**Table 1:** Sociodemographic and juridical-penal characteristics of the sample (*n* = 283).

		%	*n*
Academic qualifications	No education	0.7%	2
	4th grade	7.1%	20
	6th grade	28.3%	80
	9th grade	27.6%	78
	12th grade	17.3%	49
	Post Secondary	12.4%	35
	Bachelor’s Degree	5.3%	15
	Master’s Degree	1.4%	4
Professional Status	Unemployed	25.4%	72
	Employee	56.5%	160
	Student	9.9%	28
	Student Worker	1.4%	4
	Retired	4.9%	14
Marital Status	Single	51.9%	147
	Married/Cohabiting	35.7%	101
	Divorced/Separated	10.7%	29
	Widowed	1.1%	3
Socioeconomic Level	Low	26.5%	75
	Medium Low	9.5%	27
	Medium	40.3%	114
	Medium High	13.8%	39
	High	4.9%	14
Type of Crime	Non-violent	47.3%	134
	Violent	31.4%	89
	Both	15.2%	43

**Table 2:** Averages, standard deviations, and percentages of the main variables.

	%	*n*	M	SD
Total ACEs			2.65	2.51
At least 1 ACEs	74.2	210		
At least 2 ACEs	58.3	165		
At least 3 ACEs	42.8	121		
4 or more ACEs	32.9	93		
Emotional Abuse	31.4	89	0.49	0.78
Physical Abuse	37.1	105	0.56	0.79
Sexual Abuse	17.7	50	0.23	0.52
Emotional Neglect	29.7	84	0.41	0.68
Physical Neglect	18.4	52	0.26	0.58
Exposure to DV	72.1	204	1.23	1.55
Family Prison	21.6	61	0.21	0.41
Family Mental Illness	17.7	50	0.18	0.38
Family Substance Abuse	35	99	0.35	0.48
Divorce/separation	21.6	61	0.31	0.46
Total PCEs			8.62	1.73
At least 1 PCEs	99.6	282		
At least 3 PCEs	97.8	277		
At least 6 PCEs	89.7	254		
At least 8 PCEs	80.3	228		
ERDS-SF Total			28.18	14.25
Strategies			5.04	2.36
Non-Acceptance			6.63	2.57
Impulse			4.31	3.16
Goals			4.19	3.19
Awareness			4.03	3.07
Clarity			3.94	3.17
Social desirability			13.28	3.65

Note. ACEs—Adverse Childhood Experiences; PCE—Positive Childhood Experiences; ERDS-SF—Emotional Regulation Difficulties Scale—Short Form.

**Table 3:** Pearson correlations.

	1	2	3	4	5	6	7	8	9
1. ACEs Total	1								
2. PCEs Total	−0.372 ***	1							
3. ERDS-SF Total	0.312 ***	−0.198 ***	1						
4. Strategies	0.234 ***	−0.112	0.655 ***	1					
5. Non-Acceptance	0.086	0.054	0.633 ***	0.520 ***	1				
6. Impulse	0.249 ***	−0.198 ***	0.861 ***	0.450 ***	0.435 ***	1			
7. Goals	0.275 ***	−0.183 **	0.900 ***	0.449 ***	0.465 ***	0.772 ***	1		
8. Awareness	0.336 ***	−0.222 ***	0.887 ***	0.447 ***	0.424 ***	0.714 ***	0.797 ***	1	
9. Clarity	0.305 ***	−0.252 ***	0.869 ***	0.442 ***	0.332 ***	0.717 ***	0.785 ***	0.825 ***	
10. SDRS-5	0.283 ***	−0.271 ***	0.189 **	0.030	−0.064	0.206 ***	0.148 *	0.260 ***	0.273 ***

Note. * *p* < 0.05; ** *p* < 0.01; *** *p* < 0.001; ACEs—Adverse Childhood Experiences; PCE—Positive Childhood Experiences; ERDS-SF—Emotional Regulation Difficulties Scale—Short Form.

**Table 4:** Regression analyses.

	*b*	SE	t	*p*	95% CI
	-	-	-	-	-
Emotional Regulation Difficulties (outcome)					
Adverse Childhood Experiences	1.452	0.353	4.111	<0.001	[0.757; 2.148]
Positive Childhood Experiences	−0.636	0.508	−1.252	0.212	[−1.635; 0.363]
Social desirability	0.364	0.234	1.556	0.121	[−0.096; 0.824]
Strategies (outcome)					
Adverse Childhood Experiences	0.220	0.060	3.651	<0.001	[0.102; 0.339]
Positive Childhood Experiences	−0.053	0.087	−0.610	0.542	[−0.224; 0.118]
Social desirability	−0.032	0.040	−0.797	0.426	[−0.110; 0.047]
Impulse (outcome)					
Adverse Childhood Experiences	0.221	0.079	2.780	0.006	[0.064; 0.064]
Positive Childhood Experiences	−0.179	0.114	−1.570	0.118	[−0.404; 0.045]
Social desirability	0.111	0.053	2.108	0.007	[0.007; 0.214]
Goals(outcome)					
Adverse Childhood Experiences	0.290	0.080	3.608	<0.001	[0.132; 0.449]
Positive Childhood Experiences	−0.151	0.116	−1.304	0.193	[−0.378; 0.077]
Social desirability	0.052	0.053	0.979	0.328	[−0.053; 0.157]
Awareness (outcome)					
Adverse Childhood Experiences	0.316	0.075	4.249	<0.001	[0.170; 0.463]
Positive Childhood Experiences	−0.144	0.107	−1.348	0.179	[−0.355; 0.067]
Social desirability	0.136	0.049	2.752	0.006	[0.039; 0.233]
Clarity (outcome)					
Adverse Childhood Experiences	0.260	0.077	3.361	<0.001	[0,109; 0.412]
Positive Childhood Experiences	−0.233	0.111	−2.099	0.037	[−0.451; −0.015]
Social desirability	0.155	0.051	3.028	0.003	[0.054; 0.255]

**Table 5:** Moderation analyses.

	*b*	SE	t	*p*	95% CI
	-	-	-	-	-
Emotional Regulation Difficulties (outcome)					
Adverse Childhood Experiences	1.453	0.354	4.103	<0.001	[0.756; 2.149]
Positive Childhood Experiences	−0.627	0.582	−1.077	0.283	[−1.773; 0.519]
Social desirability	−0.006	0.191	−0.031	0.975	[−0.382; 0.370]
Interaction	0.364	0.235	1.553	0.122	[−0.098; 0.826]
Strategies (outcome)					
Adverse Childhood Experiences	0.220	0.060	3.638	<0.001	[0.756; 2.149]
Positive Childhood Experiences	−0.070	0.099	−0.708	0.480	[−1.773; 0.519]
Social desirability	0.012	0.033	0.361	0.719	[−0.382; 0.370]
Interaction	−0.033	0.040	−0.815	0.416	[−0.098; 0.826]
Impulse (outcome)					
Adverse Childhood Experiences	0.221	0.080	2.773	0.006	[0.064; 0.377]
Positive Childhood Experiences	−0.187	0.131	−1.428	0.154	[−0.444; 0.071]
Social desirability	0.005	0.043	0.122	0.903	[−0.079; 0.090]
Interaction	0.110	0.053	2.094	0.037	[0.007; 0.214]
Goals(outcome)					
Adverse Childhood Experiences	0.291	0.081	3.605	<0.001	[0.132; 0.449]
Positive Childhood Experiences	−0.135	0.133	−1.015	0.311	[−0.396; 0.127]
Social desirability	−0.011	0.044	−0.252	0.801	[−0.097; 0.075]
Interaction	0.053	0.053	0.990	0.323	[−0.052; 0.158]
Awareness (outcome)					
Adverse Childhood Experiences	0.318	0.075	4.255	<0.001	[0.171; 0.465]
Positive Childhood Experiences	−0.089	0.123	−0.726	0.469	[−0.331; 0.153]
Social desirability	−0.037	0.040	−0.926	0.355	[−0.117; 0.042]
Interaction	0.138	0.050	2.800	0.006	[0.041; 0.236]
Clarity (outcome)					
Adverse Childhood Experiences	0.261	0.077	3.369	<0.001	[0.108; 0.413]
Positive Childhood Experiences	−0.193	0.127	−1.519	0.130	[−0.443; 0.057]
Social desirability	−0.027	0.042	−0.643	0.521	[−0.109; 0.055]
Interaction	0.157	0.051	3.057	0.003	[0.056; 0.257]

## Data Availability

The datasets generated during and/or analyzed during the current study are not publicly available due to confidentiality of the data, but are available from the corresponding author on reasonable request. The data presented in this study are available on request from the corresponding author due to privacy issues.
